# Exploring adaptation responses of maize to climate change scenarios in southern central Rift Valley of Ethiopia

**DOI:** 10.1038/s41598-023-39795-y

**Published:** 2023-08-09

**Authors:** Daniel Markos, Walelign Worku, Girma Mamo

**Affiliations:** 1 School of Plant and Horticultural Sciences, Hawassa University, P.O.Box-05, Hawassa, Ethiopia; 2 Ethiopian Institute of Agricultural Research, P.O.Box-2003, Addis Ababa, Ethiopia

**Keywords:** Climate sciences, Environmental sciences

## Abstract

In this study, we assessed responses of adaptation options to possible climate change scenarios on maize growth and yield by using projections of 20 coupled ensemble climate models under two representative concentration pathways (RCPs) 4.5 and 8.5 by means of a DSSAT model. Growth and yield simulations were made across present and future climate conditions using the hybrid maize variety (Shone). Subsequently, simulated yields were compared with farmer’ average and on-farm trial yields. Results showed that on-farm trial yield (5.1–7.3 t ha^−1^) lay in between farmers’ average yield (2.9–5 t ha^−1^) and water-limited potential yield (6.3–10.6 t ha^−1^). Maize yields achieved in farmers’ fields are projected to decline towards mid-century and further towards the end of the century regardless of the adaptation options compared with baseline in low potential clusters. Results of a combination of adaptation options including February planting, use of 64 kg ha^−1^ N and conservation tillage provided yield advantage of 5.8% over the 30 cm till under medium GHGs emission scenario during mid-century period at Shamana. Mulching with 5 t ha^−1^ was projected to produce a 4–5% yield advantage in the Hawassa cluster during the mid-century period regardless of changes in tillage or planting window. Under a high GHGs emission scenario, over 13.4% yield advantage was projected in the Bilate cluster due to conservation tillage and June planting during the mid-century period. In the Dilla cluster, the use of 10 t ha^−1^ mulch, conservation tillage and early planting (February) would result in a 1.8% yield advantage compared with the control either in medium or high GHGs emission scenarios. Thus, the most promising and least risky practices among simulated strategies were the use of nitrogen and mulching in combination with tillage or planting date adjustment. However, adaptation options remained least promising and highly risky if not integrated with mulching or nitrogen use. Hence, the negative impacts of future climate change and subsequent yield gaps would be reduced by optimizing the application of nitrogen, mulch and their interaction with planting date and tillage in high and low potential areas of maize production.

## Introduction

Maize is the major food, feed, and industrial crop globally, and the leading staple food crop in many developing countries. With 1147 million metric tons (MMT) produced on 193.7 million hectares globally^1^, maize is the leading cereal in terms of production. However, the average maize yields have declined globally by about 3.8% over the last decades due to climate related shocks^2^. Maize potential and attainable yields were estimated to reduce by 2.1% and 8.0%, respectively from 1961 to 2009 due to climate change^3^. Maize yield may see a decline of 24% by late century, with declines becoming apparent by 2030, when the global cereal demand for food and animal feed alone is expected to increase by 50% higher than in 2000^4^ and there is another projection of 60% increase in food demand by 2050^5^. This is mainly due to shifts in rainfall patterns and temperatures regimes arising from increases of CO_2_, which would probably make growing food crops more difficult to in the tropics.

Climate change increases uncertainty in food production by undermining efforts of mitigating threats to global food security in the 21st century. Previous studies have showed that the negative impacts of climate change on maize yield were mainly associated with the warming and increased drought frequency during the growth periods and reduction of farm lands eventually reducing production and creating yield gaps^6–8^. In Sub-Saharan Africa, gaps between the potential and the actual maize yields are caused by biotic (diseases, weeds, insects and other arthropod pests), abiotic (drought and nutrient deficiencies), or socioeconomic (adoption, input and market) constraints^9–11^ leading to recurring food shortages^12^. This wider yield gaps should be filled by matching agronomic adaptation options and seed genetic improvement that counterfeit the ongoing climate change and combat food insecurity challenges^3,13–15^.

The likely response of cultivar or management response to the impacts of climate change on crop yield can be determined through the field experimentation and simulation modeling research. A number of simulation models are being used for fast-tracking crop–environment processes, in filling gaps of field experiments, by minimizing the expensive and time-consuming agronomic trials, and for analyzing the yield gaps between the potential and actual productivity of various crops under the changing climate and in exploring both indigenous and modern adaptation responses^16–20^. Among the models, Decision Support System for Agricultural Technology Transfer (DSSAT) presents an important opportunity for scoping the short-duration field experimental results into the long-term, by using the current, as well as the projected climate and soil information^21,22^. DSSAT model has been tested and used in the environments of Sub-Saharan Africa including Ethiopia^23^, calibrated and tested for different maize cultivars^9^ and recommended for further use by these authors. The cumulative thermal time from seedling emergence to the end of the juvenile phase (P1), and from silking to physiological maturity (P5) are among the most important determinants of maize yield which could be used in yield prediction^21,22^. Thus, the germination process of maize benefits from abundant solar radiation and an increased temperature within the optimum range of crop’s heat requirement^24–26^. Hence, the DSSAT simulation assumes that reasonable increase of the values of the P1 and P5 would mitigate the negative impacts of future climate change on maize yields, implying that cultivars with longer growing season should be developed in order to cope with heat load and benefit from warmer future climate^13^.

Planting date adjustments and fertilizer applications conducted following forecasted rainfall are among the most widely studied responses of adapting to climate risks^9,13,26–29^. This is because optimal planting dates and fertilizer regimes could offset adverse impacts of climate change by matching crop growth with changing temperature and rainfall distribution. Modifications of mulching or tillage rates and methods have also been used as adaptation options^30,31^, but their impact on crop production depends on climate^32^, soil texture, and slope^33,34^. Mulching would reduce soil water evaporation and run-off^35^, increase topsoil organic matter and improved near-surface soil aggregate properties^36^, potentially bearing positive effects on crop productivity^37^. Khurshid et al.^31^ also attributed a yield contribution of about 20% to tillage related changes. However, adaptation options like mulching had the opportunity cost of nitrogen immobilization in the short run in that, for every 10 units of carbon sequestered in the soil; there is a need to immobilize one unit of nitrogen^38^. Similarly both N fertilization and residue retention significantly enhance ammonia (NH_3_) and nitrous oxide (N_2_O) emissions^39^. This calls for splitting application of N fertilizer to improve the temporal synchronicity between crop N demand and soil N availability, thereby increasing crop yield and reducing residual soil nitrogen and its environment risk^26,40^. Hence, there is a need to consider fertilizer rating and timing to meet the nutrient demands of crop growth and avoid nutrient loss when crop biomass decreases due to water and temperature stress over time under climate risks^13,15^. Thus, adaptation options have their own management requirements that vary with local natural resource endowment rendering them location specific, which in turn calls for optimization of adaptation options across different agro-ecologies.

Southern central Rift Valley of Ethiopia is one of the most vulnerable regions to climate risks, where increased temperatures and changes in rainfall patterns have been altering the evapotranspiration rate and reducing water availability for maize crop^41,42^. Moreover, soil conditions have been changing due to the associated land degradation processes over the years and hence the old management recommendations may not be efficient, thus making generation of new adaptation options a necessity. Hence, quantifying climate related risks and adapting agriculture to the changing climate is inevitability daunting challenge in the study area. Thus, exploring effective adaptation strategies and measuring their effect on the performance of maize crop is crucial to improving maize production under future climate scenarios. However, past climate change studies carried out in Ethiopia have been limited to assessment of impacts of crop production without accounting for potential adaptation measures^43,44^. Moreover, information on climate related yield variability, climatic yield potentials and the magnitude of yield gaps due to nutrient and water limitation, and their associated management was scanty^9^. In addition, the difference between maize yield potential and the actual yield varies from place to place. Therefore, the objective of this study was (1) to analyze existing yield gaps using DSSAT model in the southern central Rift Valley of Ethiopia, and (2) to explore potential adaptation strategies and develop agronomic recommendations that reduce the negative impacts of future climate risks on maize yield in the study areas.

## Materials and methods

### Description of the study area

**Figure 1:**
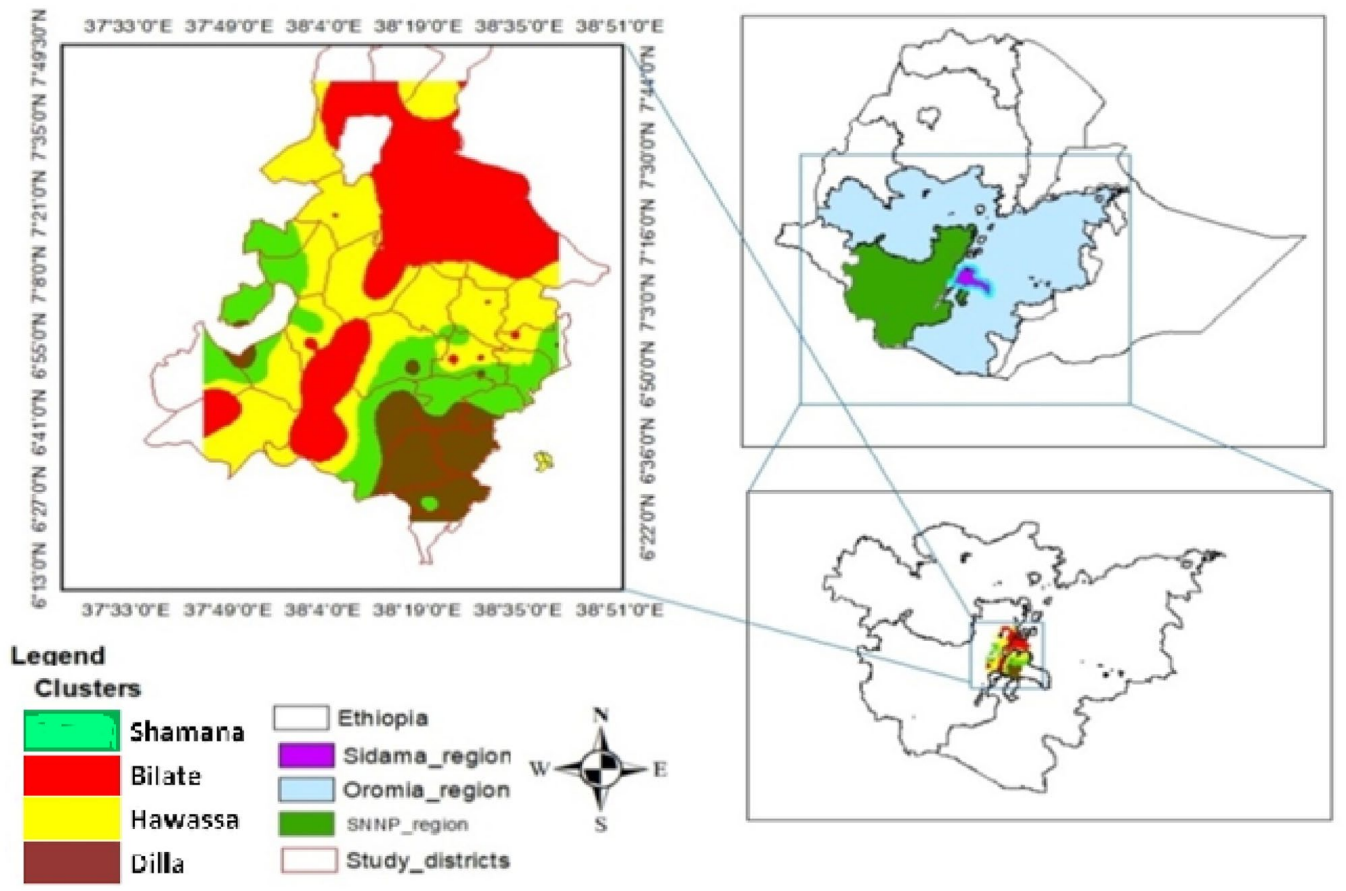
The study area map with the four distinct clusters (Map of the study area with four clusters was created by using free version of QGIS version 3.32, https://www.qgis.org/en/site/forusers/download.html/).

This study was carried out in southern plains of central Rift Valley of Ethiopia located between 6.38 latitudes in Dilla area (South) to 7.72 latitudes in Bulbula area (North). The Western margin corresponds with 37.75 longitudes (Wolita sodo) to 38.68 latitudes in Woteraresa (East). This covers an area of 1,021,332 ha (Fig. 1). The study area has been grouped using prnicipal component and cluster analysis of physiographic, climatic and topographic attributes into four distinct clusters each having a number of districts represnting the area as shown in Markos et al.^45^.

### Historic climate

The long term average annual rainfall for Shamana, Bilate, Hawassa and Dilla clusters is 972.0 ± 97.8 mm, 758.7 ± 125.8 mm, 953.9 ± 57.5 mm and 1298.2 ± 70.4 mm^42^, respectively. About 33.0 and 42.9% of the average annual rainfall in Shamana cluster is received during ONDJ and JJAS period, respectively. About 36.0 and 37.2% of the average annual rainfall in Bilate cluster is received during FMAM and JJAS period, respectively. About 33.0 and 42.9% of the average annual rainfall in Hawassa cluster is received during ONDJ and JJAS period, respectively. About 35.3 and 33.3% of the average annual rainfall in Dilla cluster is received during FMAM and JJAS period, respectively^45^. Shamana cluster have a mean minimum and maximum temperature of 15.5 and 26.4 °C, respectively. Similarly Bilate, Hawassa and Dilla clusters have mean minimum and maximum temperature of 19.3 and 28.4 °C, 14.2 and 27.1 °C and 15.8 and 28.0 °C, in respective areas^42^.

### Soil

In Shamana cluster, soils are developed from volcanic sedimentary to lacustrine deposits and have relatively higher clay and dominantly belong to *Vertic Luvisols*, *Eutric Cambisols*, and *Chromic Vertisols* based on FAO soil classification^45^. In Bilate cluster, the most dominant soil is riverine or lacustrine alluvium derived from basalt, ignimbrite, lava, or ash with dark brown loamy coarse sands and sandy loams with often calcareous subsoil, and belong to *Calcaric Fluvisol*, *Orthic Andosol*, *Ortic Phaeozems*, and *Chromic Luivisols*. In Hawassa cluster, the dominant soils are *Vitric Andosols*, *Eutric Cambisols*, and *Leptosols*. The soils had lacustrine and pyroclastic deposits of sands and silts, interbedded with pumice. In Dilla cluster the soils are originated from basalt rocks, and had deep red-dish to brown clayey to clay loam texture classified as *Haplic Luvisols* and *Chromic Vertisols*. Low bulk density and weak structure of soils in Bilate and Hawassa clusters render them vulnerable to erosion even on gentle slopes compared with those in Shamana and Dilla clusters (Markos et al.^57^). The soils of study area at Shamana, Bilate, Hawassa and Dilla had organic carbon content of 3.99, 3.61, 4.33 and 4.96%, respectively.

### Farming system

The Shamana cluster stands for high-land transitional moist areas with upper mid-altitude category^46^ or highland sub-humid areas^47^. The Shamana cluster had enset/maize based potato farming system in Shamana, maize based tef farming system in Shone and maize based faba bean farming system in Bitena areas. The cluster is characterized by intermediate to high altitudes and cooler temperatures allowing December–January planting of maize, and double cropping of the second crop usually starting in July. The crop residues have been used for cattle feed. The Bilate cluster is located in the valley bottom of the rift valley attaining the lowest altitude and hottest temperature among the clusters; sorghum/finger millet/maize production begins in April–May period in this cluster. The Bilate cluster represented maize production agro-ecology with large number of livestock termed as semiarid lowlands of central Rift Valley or dry mid-altitudes^46^ and low moisture areas^47^. Currently, the Hawassa cluster is known for rain-fed maize-based production systems and modest livestock rearing^9^. It possesses intermediate altitude and dominant maize cropping allowing common beans for intercropping in most cases. The Hawassa cluster was one of the sub-moist mid altitude areas in central Rift Valley which is also called mid-altitude sub-humid area^47^. Finally low to mid- altitude areas adjacent to mountain chains of Sidama and Gedeo, also known for indigenous coffee, enset and livestock based agroforestry practices, were grouped into Dilla cluster. The Dilla cluster is represented as humid tropics^48^, moist low to mid-altitude areas^46^, and as lowland sub-humid areas^47^.

### Data sources

Average farmers’ yield representing farmer managed maize plots was obtained by using questioniare interview from 60 farmers per district representing a given cluster. This has been added and then divided to obtain the average average farmers yield per cluster. Furthermore, the obtained yield has been trianglated with reports of district offices of agriculture for each cluster. Next on farm trial yields, maize yield from researcher designed researcher managed trials, were collected from reports of researchers^49–56^. The model was calibrated using historic weather data of thirty years (1991–2020), field measured values of crop parameters and soil properties during 2011–2017 cropping season. Thus, calibration was done using experimental data for the period between 2011 and 2017 whereas validation was done using experimental data for the period between 2016 and 2018 period. Using genetic coefficients already calibrated and evaluated for hybrid maize variety *Shone*^57^, and current and future climate data downscaled from contrasting multimodel ensembles of 20 Global Climate Models (GCMs) over Representative Concentration Pathways (RCP) 4.5 and 8.5^58^, maize yields were anticipated. Thus, the water-limited potential yield for each cluster was obtained through simulation using DSSAT 4.8, where water was switched off under baseline (current) scenareo using the hybrid maize variety (*Shone*) and commonly practiced management practices. Under future climate scenarios, the maangment practices were alttered as designed in the adaptation strategies section of this manuscript and the water remained switched off for the same hybrid maize variety.

### Yield gap (Yg) analysis

Yield gap analysis was performed at three levels following procedures established by Kassie et al.^59^ as shown below in Eqs. (1, 2, 3):

1 $${\text{Yield }}\;{\text{gap }}\,{\text{I}}\; = \;Yb - {\text{Ya}}$$

2 $${\text{Yield }}\;{\text{gap}}\;{\text{ II}}\; = \;Yc - {\text{Yb}}$$

3 $${\text{Yield }}\;{\text{gap}}\;{\text{ III}}\; = \;Yc - {\text{Ya}}$$

where Yield Gap I—Gap between simulated water-limited potential yield and average farmers’ yield.

Yield Gap II—Gap between water-limited potential yield and on-farm tal yield.

Yld Gap III—Gap between on-farm trial yield and average farmers yield.

Yb = Simulated water limited potential yield.

Ya = Average farmers’ yield.

Yc = On-farm trial yield.

### Adaptation strategies

Four adaptation options each having different levels of strategies were explored as in Table 1.

**Table 1:** Adaptation strategies used in the simulated experiment.

Factor	Cluster	Levels
Planting date	Shamana:	December 1st, January 1st (control)^c^ and February 1st
	Bilate:	April 1st, May 1st (control) and June 1st
	Hawassa:	March 1st, April 1st (control) and May 1st
	Dilla:	February 1st, March 1st (control) and April 1st
Nitrogen^a^	All	0 (control), 64 and 128 kg ha^−1^
Tillage	All	zero till or conservation agriculture 30 cm till or traditional oxen plow (control)
Mulching^b^	All	0 (control), 5 and 10 t ha^−1^

^a^The nitrogen treatments were adjusted for each of Shamana, Bilate, Hawassa and Dilla clusters in two splits (1/3 of nitrogen applied 15 days after planting and 2/3 of nitrogen applied between 40 and 45 days after planting at knee height).

^b^Mulching applied in a single dose prior a month of planting.

^c^The control is a baseline scenario representing conventional 30 cm till that is unfertilized and unmulched, which represents the classic practices in resource-limited smallholder farming systems.

### Analysis of variance (ANOVA)

The output of DSSAT model was statistically analyzed using the analysis of variance (ANOVA) technique to evaluate the impact of climate change on maize production. Accordingly the four management and their levels collectively 54 management strategies (three nitrogen rates × three planting dates × three mulch rates × two tillages) were compared using Randomized Complete Block Design in factorial combination of the treatments. The year effect, which has 30 levels, was used as replications (blocks) as in the DSSAT software because the maize yield in 1 year under a given treatment was not affected by another year. Since each simulation year had unpredictable weather conditions, the soil organic carbon and moisture were reset each year, formal randomization of simulation years was not needed^16,60^. The ANOVA was calculated using the SAS software package and treatments averages were separated using least significance difference (LSD) at 5% level of probability wherever difference between averages exist.

### Estimation of yield changes under different scenarios

The biophysical impact of climate change across southern central rift valley of Ethiopia was obtained by calculating changes in crop yield between the current and the proposed adaptation options for hybrid maize variety called *Shone*. Then the changes were calculated for each of early, mid and late century period relative to the current practice for the respective GHGs emission scenarios. Accordingly impacts of adaptation options were computed based on^61^ given in Eq. (4) as shown below.

4 $${\text{Yield }}\;{\text{change }}\left( {\text{\% }} \right) = \frac{{\left( {{\text{Yanticipated}} - {\text{Ycontrol}}} \right){*}100}}{{{\text{Ycontrol}}}}$$

where Y anticipated—simulated yield under new adaptation option.

Y control—simulated yield under control.

### Ethics approval and consent

Hawassa University hosts graduate students from higher educational institutions and agricultural research centers, who are publicly allowed to gather, analyze and report local data to global scientific communities. All methods of experimental research and field studies used in this study comply with relevant institutional, national, and international guidelines and legislations. The study has been exempted from the requirements of the human subject protections regulations.

## Results

### Average farmers’ yield and yield gaps

**Figure 2:**
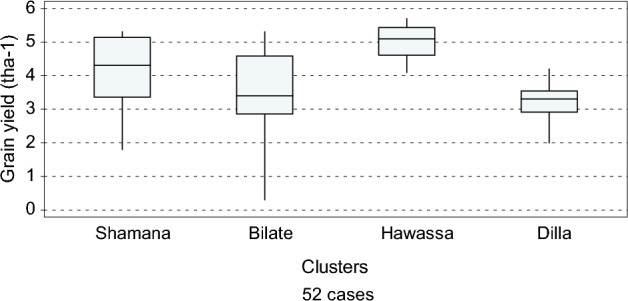
Box and Whisker plot of average farmers’ yield (t ha^−1^) across the four clusters.

The result showed that the average of thirteen years farmers’ average yield was high at Hawassa (5 t ha^−1^) and Shamana (4.2 t ha^−1^), but low at Bilate (3.4 t ha^−1^) and Dilla (3.2 t ha^−1^) (Fig. 2). The standard deviations of maize yield were highest at Bilate depicting higher variability across years, which is manifestation of environmental vulnerability of the cluster. This showed that the locations Hawassa and Shamana had high potential for maize production compared with those of Dilla and Bilate, which could be due to climatic and agro-ecological suitability of the clusters.

The average actual maize yield (2.9–5 t ha^−1^) of districts located within a given cluster formed yearly average farmers’ yield, which is the lowest compared with experimental (5.1–7.3 t ha^−1^) and water limited potential yield (6.3–10.6 t ha^−1^) (Table 2). Farmers' yield is 46–54% of on-farm experimental yield and in turn on farm experimental yield was 23.5–45.2% of the water limited potential yield in southern central rift valley of Ethiopia, which was twice the gap between research and farmers’ average yield. Farmers’ yield was 46–54% of on-farm experimental yield mainly due to lack of improved seeds/fertilizers, lack of credit, poor extension services, and poor field management for weeds, insect pests and diseases. This calls for integrated crop management, credit to farmers, timely supply of inputs, research and extension collaboration to transfer the new technologies trough training, demonstrations, field visits and advice by extension agents etc. On-farm trial yield (5.1–7.3 t ha^−1^) lied in between farmers’ average yield (2.9–5 t ha^−1^) and water limited potential yield (6.3–10.6 t ha^−1^), which is attributed to higher use of fertilizers, intensive weed, insect pest and disease control. This agrees with previous findings of^62^ that states average yields in rain-fed systems are commonly 50% or less of yield potential, suggesting ample room for improvement, However, the potential yields were lower than those reported by some authors^63,64^, who stated 12.5 t ha^−1^ simulated potential yield for maize yield in Ethiopia. The yield obtained from experimental field is constrained by rainfall and soil water characteristics, and can be enhanced through use of increased investment in agricultural research infrastructure including molecular laboratories, tissue culture facilities, irrigation structures, nut tunnels, screen houses, etc), policy alignment with research and development to enhance technology generation and transfer and skilled research personnel. The water limited potential yield reported in this study was lower than previous report by other scholars due to increased uncertainty in growing season weather in the study clusters, and should be enhanced by increasing genetic yield potential, using heat tolerance and high harvest index cultivars, which requires breakthroughs in genetic improvement research. The result of this study is in agreement with those of Kassie et al.^59^ who elaborated actual yields to be 28–30% of the water-limited potential yield and 44–65% of on-farm trial yields in relative terms while the on-farm trial yields were 50–73% of the water-limited potential yield.

**Table 2:** Comparison of farmers yield, experimental and potential yield in the study area.

Clusters	Average farmers’ yield (t ha^−1^) Ya	On-farm trial yield (t ha^−1^) Yb	Water stressed potential yield (t ha^−1^) Yc	Yield gap 1 (t ha^−1^) (Yb–Ya)	Yield gap 2 (t ha^−1^) (Yc−Ya)	Yield gap 3 (t ha^−1^) (Yc−Yb)
Shamana	3.9–4.5	6.0–7.2	8.1–8.7	2.2–3.1	4.3–4.6	1.5–2.1
Bilate	3.0–3.8	5.7–6.0	6.6–7.1	2.6–2.6	3.5–3.7	0.9–1.1
Hawassa	4.9–5.2	6.8–7.3	9.8–10.6	2.0–2.3	5.0–5.6	3.0–3.3
Dilla	3.1–3.4	5.1–5.2	6.3–6.8	1.9–2.2	3.4–3. 6	1.2–1.6

### Analysis of variance for projected maize yields against adaptation options

The mean square of error (MSE) values depicted that use of mulch (P < 0.001), nitrogen (P < 0.001), and tillage × planting date (P < 0.001) affected maize yield significantly during baseline scenario at Shamana cluster (Table 3). The same trend was projected across medium and high emission scenarios during early, medium and late century for these practices at Shamana. The interactions of tillage × planting date × nitrogen or mulching would affect maize yield significantly (P < 0.05) across high emission scenario during early, mid and late century at Shamana. At Bilate cluster, use of mulch (P < 0.001), tillage × nitrogen × planting date (P < 0.001), and tillage × mulching × planting date (P < 0.001) affected maize yield significantly during baseline scenario (Table 3). This trend was maintained across medium and high emission scenarios during early, medium and late century at Bilate. Moreover, use of nitrogen affected maize yield significantly (P < 0.05) across RCP 4.5 and RCP 8.5 during mid and late century at Bilate. Lack of response of maize yield to application of nitrogen at Bilate to during baseline and early century could be attributed to sub-optimal moisture in the cluster.

**Table 3:** MSE for grain yield across medium and high GHGs emission scenarios during early, medium and late-century period.

Source	DF	Baseline	4.5E	4.5M	4.5L	8.5E	8.5M	8.5L
Shamana								
TL	1	1417831 ns	8,883,236**	2,931,639 ns	479,124 ns	451,360 ns	121,680 ns	220,422 ns
PD	2	1325 ns	0 ns	0 ns	0 ns	0 ns	0 ns	0 ns
RS	2	8,010,394***	5,917,969**	12,770,000**	4,310,605*	5,804,935*	7,863,453*	11,750,000***
N	2	89,810,000***	5,546,841**	9,587,199***	5,226,276**	4,176,855*	5,447,042*	9,275,942***
TL × N	2	4,194,922 ns	415,597 ns	454,032 ns	771,927 ns	1,216,402 ns	1,081,547 ns	314,971 ns
PD × N	4	12,080,000 ns	12,271 ns	4062 ns	4598 ns	6523 ns	5167 ns	4879 ns
TL × PD	2	5,415,203***	35,530,000***	11,730,000**	1,916,498 ns	1,805,441 ns	486,720 ns	881,690 ns
TL × RS	2	69,847 ns	253,599 ns	430,449 ns	1,016,140 ns	1,191,142 ns	2,087,197 ns	565,771 ns
PD × RS	4	1325 ns	0 ns	0 ns	0 ns	0 ns	0 ns	0 ns
TL × PD × N	4	9,757,320 ns	1,167,741 ns	1,300,057 ns	2,707,256*	5,592,551**	4,379,658*	1,469,499 ns
TL × PD × RS	4	335,823 ns	1,014,396 ns	1,721,798 ns	4,064,560**	4,764,569*	8,348,788**	2,263,086*
CV (%)		20.1	17.93	17.5	20.81	20.81	24.91	28.06
Bilate								
TL	1	612,599 ns	2136 ns	675,433 ns	2,578,259*	35,575 ns	467,284 ns	21,901 ns
PD	2	646,265 ns	0 ns	0 ns	0 ns	0 ns	0 ns	0 ns
RS	2	4,165,358***	577,053 ns	7,698,185**	740,854 ns	2,743,552*	5,498,059***	2,477,716*
N	2	1,382,269 ns	2,449,633*	7,949,970***	567,101 ns	452,378 ns	5,507,704***	2,255,397**
TL × N	2	550,119 ns	554,136 ns	750,282 ns	95,413 ns	423,856 ns	802,281 ns	166,074 ns
PD × N	4	1,291,931 ns	308,768 ns	2905 ns	2481 ns	108,144 ns	172 ns	4378 ns
TL × PD	2	668,134 ns	8544 ns	2,701,730*	10,310,000***	142,301 ns	1,869,135*	87,605 ns
TL × RS	2	177,309 ns	66,945 ns	849,092 ns	109,992 ns	90,607 ns	817,235 ns	141,514 ns
PD × RS	4	2,315,043 ns	0 ns	0 ns	0 ns	0 ns	0 ns	0 ns
TL × PD × N	4	6,063,529***	1,624,869 ns	3,207,081***	437,888 ns	1,544,344 ns	3,116,157**	567,180 ns
TL × PD × RS	4	4,562,339***	267,778 ns	3,396,367**	439,968 ns	362,426 ns	3,268,940***	566,058 ns
CV (%)		18	13	13	13	14	11	15
Hawassa								
TL	1	1808 ns	2,534,363 ns	878,541 ns	7676 ns	118,785 ns	800,184 ns	714,751 ns
PD	2	0 ns	0 ns	0 ns	0 ns	0 ns	0 ns	0 ns
RS	2	18,460,000***	6,470,965***	1,726,119*	2,051,894*	5,780,444**	735,741***	2,014,028*
N	2	13,110,000***	1,679,010***	1,775,618***	1,992,634***	571,821***	1,368,890***	470,853***
TL × N	2	368,281 ns	754,946 ns	503,504 ns	382,047 ns	211,259 ns	42,500 ns	969,905 ns
PD × N	4	594 ns	229 ns	59 ns	332 ns	1827 ns	933 ns	512 ns
TL × PD	2	7231 ns	10,140,000***	3,514,163 ns	30,706 ns	475,141 ns	3,200,736 ns	2,859,002**
TL × RS	2	270,938 ns	418,989 ns	35,389 ns	416,903 ns	257,563 ns	65,108 ns	722,046 ns
PD × RS	4	0 ns	0 ns	0 ns	0 ns	0 ns	0 ns	0 ns
TL × PD × N	4	1,364,096 ns	2,794,223**	2,012,712**	1,459,045*	888,676 ns	161,857 ns	3,944,794**
TL × PD × RS	4	1,083,752 ns	1,675,957 ns	141,557 ns	1,667,613*	1,030,253 ns	260,433 ns	2,888,185***
CV (%)		21.46	13.49	13.87	11.16	13.65	9.74	13.93
Dilla								
TL	1	1,212,397 ns	85,353 ns	817,367 ns	2,075,512*	1,759,822	683,165 ns	65,098 ns
PD	2	0 ns	0 ns	0 ns	0 ns	0	0 ns	0 ns
RS	2	8,383,447*	5,668,811***	721,872 ns	659,104**	15,750,000***	10,970,000***	5,746,542**
N	2	7,686,620*	5,707,874*	575,568 ns	6,776,404**	15,470,000***	10,700,000***	5,610,019**
TL × N	2	417,261 ns	1,476,535 ns	225,753 ns	269,014 ns	1,576,116	171,660 ns	1,313,885*
PD × N	4	4144 ns	407 ns	732 ns	194 ns	1316	668 ns	62 ns
TL × PD	2	4,849,589 ns	341,411 ns	3,269,467*	8,302,048***	7,039,288**	2,732,658*	260,393 ns
TL × RS	2	375,278 ns	1,462,564 ns	376,235 ns	240,100 ns	1,531,834	173,733 ns	1,305,735*
PD × RS	4	0 ns	0 ns	0 ns	0 ns	0	0 ns	0 ns
TL × PD × N	4	1,646,290 ns	5,805,778*	953,434 ns	961,438 ns	6,332,834**	690,019 ns	5,151,820**
TL × PD × RS	4	1,501,114 ns	5,850,255***	1,504,940 ns	960,399 ns	6,127,334***	694,933 ns	5,222,938**
CV (%)		13.09	11.52	11.34	9.79	9.67	11.55	11.66

TL—tillage, N—nitrogen rate, PD—planting date and RS refers to residue application (mulching), 4.5E, 4.5M and 4.5L represent medium GHGs emission scenario during early, mid and late century; 8.5E, 8.5M and 8.5L show high e GHGs mission scenario during early, mid and late—century.

*, **, ***Denotes presence of significant difference at 0.05, 0.01 and 0.001% level of probability; ns denotes absence of significant difference at 0.05% level of probability.

At Hawassa cluster, use of mulch (P < 0.001) and nitrogen (P < 0.001) affected maize yield significantly during baseline scenario (Table 3). The same trend was maintained across medium and high emission scenarios during early, medium and late century. During mid and late century, the interaction of tillage × nitrogen × planting date or tillage × mulching × planting date were projected to affect maize yield significantly (P < 0.05) across RCP 4.5 and RCP 8.5 at Hawassa. At Dilla cluster, use of mulch (P < 0.001) and nitrogen (P < 0.001) affected maize yield significantly during baseline scenario (Table 3). This trend was projected to continue across medium and high emission scenarios during early, medium and late century. Across RCP 4.5 and 8.5, interaction of tillage × nitrogen × planting date or tillage × mulching × planting date was projected to affect maize yield significantly (P < 0.05) during early, mid and late century at Dilla.

### Trends of yield change in response to adaptation strategies

#### Tillage as adaptation options

**Figure 3:**
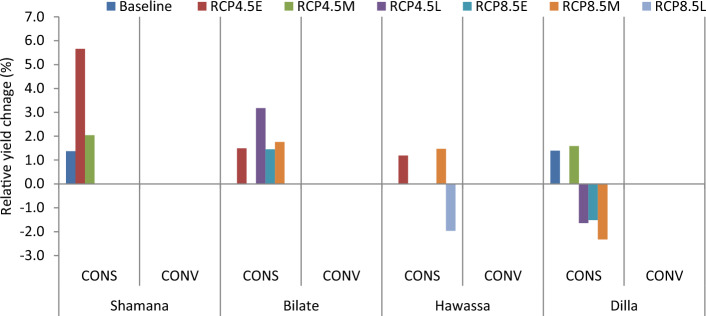
Change in grain yield (%) of shone variety in response to conservation tillage relative to traditional tillage during early, mid and late-century across baseline, RCP 4.5 and 8.5 emission scenario at Shamana, Bilate, Hawassa and Dilla clusters, southern central Ethiopia.

**Figure 4:**
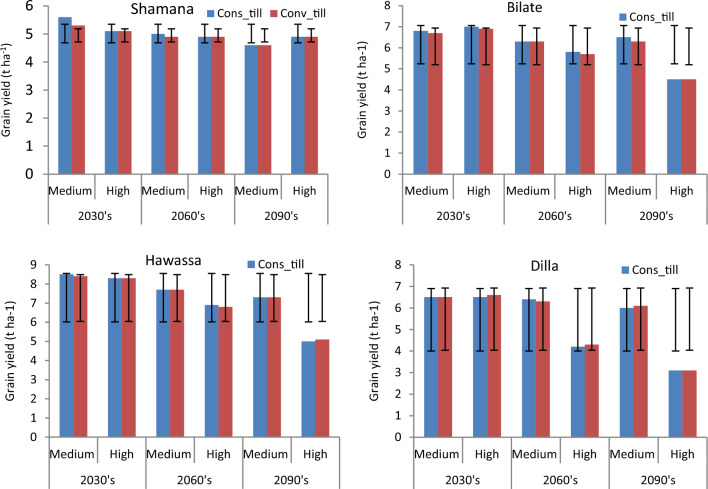
Maize yields (t ha^−1^) and error bars with standard deviation due to change in tillage practices under medium and high GHGs during early, mid and late century period across Shamana, Bilate, Hawassa and Dilla clusters.

The projection of maize yield (8.5 t ha^−1^) is highest at Hawassa due to conservation tillage across medium GHGs emission scenario during early century. The lowest grain yield of 3.1 t ha^−1^ was projected in Dilla due to 30 cm till or conventional tillage across high GHGs emission scenario during late century. Considering baseline scenario, conservation tillage is projected to increase maize yield by 1.4% in Shamana and Dilla clusters. No yield change is projected at Bilate and Hawassa under respective baseline scenarios. Maize grain yield increased by 5.7 and 2% during early and mid-century, respectively due to conservation tillage under medium GHGs emission scenarios at Shamana compared with conventional tillage (Figs. 3 and 4). However, across high GHGs emission scenarios during early, medium and late-century, and medium GHGs emission scenario during late-century changing tillage changes to conservation tillage will not be able to counteract the negative effects of climate change on maize crop at Shamana.

At Bilate, conservation agriculture produced 1.5 and 3.1% higher yields compared with conventional tillage under medium GHGs emission scenario during early and medium-century. Moreover, there will be yield advantage of 1.4 and 1.8% under high GHGs emission scenario during early and mid-century, respectively. During late-century, there was not any advantage due to alterations in tillage at Bilate. At Hawassa, only 1.2 and 1.5% yield increments were anticipated due to conservation tillage under medium and high GHGs emission during early and mid-century, respectively. There was 2% yield decline due to conservation tillage compared with conventional tillage at Hawassa under high GHGs emission scenario during late-century. At Dilla, 1.6% grain yield advantage was foreseen due to conservation tillage compared with conventional tillage under medium GHGs emission scenario during mid-century period. However, changing tillage practices from conventional to conservation resulted in 1.5 and 2.3 yield decrease under high GHGs emission scenarios during early and mid-century, respectively. The yield decline due to conservation tillage was also 1.6% under medium GHGs emission scenario during late-century at Dilla.

#### Planting dates as adaptation response

**Figure 5:**
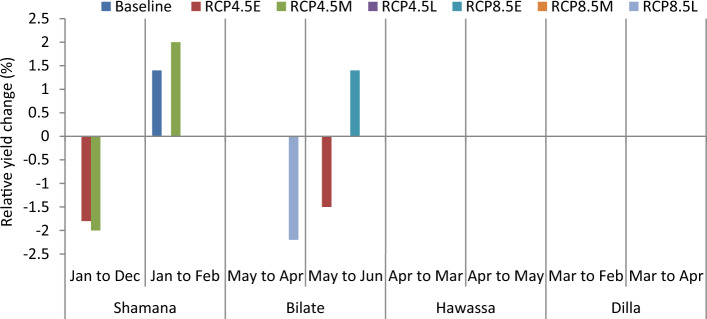
Change in grain yield (%) of shone variety in response to planting date relative to control during early, mid and late-century across baseline, RCP 4.5 and 8.5 emission scenario at Shamana, Bilate, Hawassa and Dilla clusters, southern central Ethiopia.

**Figure 6:**
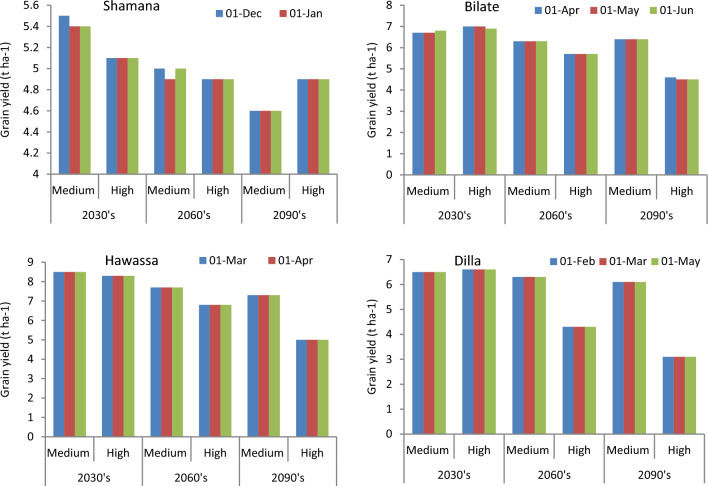
Maize yields (t ha^−1^) and error bars with standard deviation due to change in planting date under medium and high GHGs during early, mid and late century period across Shamana, Bilate, Hawassa and Dilla clusters.

The projection of maize yield (8.5 t ha^−1^) is highest at Hawassa due to April planting across medium GHGs emission scenario during early century. The lowest grain yield of 3.1 t ha^−1^ is projected in Dilla due to February planting across high GHGs emission scenario during late century. Shifting planting window backwards (eg. from January to December in Shamana, from May to April in Bilate, from April to March in Hawassa or from March to February in Dilla) did not show any yield advantage under baseline conditions. Thus, an early sowing date wouldn’t be conducive for maize production under baseline scenario. However, shifting planting window from January to February would present 1.4% yield advantage under baseline conditions in Shamana cluster (Figs. 5 and 6). Shifting planting dates from January to December resulted in 1.8 and 2% grain yield decline under medium GHGs emission scenarios during early and mid-century period, respectively in this location. Conversely, shifting planting period to February would result in 2% yield advantage under medium GHGs emission scenario during mid-century period in Shamana.

At Bilate, shifting planting window from May to June would result in 1.5% grain yield penalty under medium GHGs emission scenario during early century period. However, there would be 1.4% yield increase under GHGs high emission scenarios due to the same forward shifting of planting window for the same duration. Shifting planting window forward or backward did not show any difference under medium and high GHGs emission scenarios during mid-century period. However April planting resulted with 2.2% yield decline under high GHGs emission scenario during late century period. Thus June planting would help to adapt climate change under high GHGs emission scenario during early century at Bilate with 1.4% yield advantage over the commonly practiced May planting. At Hawassa and Dilla, the DSSAT model did not simulate any gains or losses in grain yield due to changes in planting window either under medium or high GHGs emission scenarios during early, mid and late century periods. Delaying planting was useful under baseline and medium GHGs emission scenarios during early century at Shamana, and under high climate emission scenario during early century at Bilate. This is mainly because delayed planting avoids the high-risk periods of heat and drought stress in the maize growth period. This agrees with some authors who attributed the advantage of delayed planting to increased precipitation during late July to early August, which resulted in higher pollination rates and kernel numbers without water stress^65,66^. However, delayed planting previously recommended by Disasa and Yan^67^ for Hawassa cluster was not validated with multiple climate ensembles considered in this study.

#### Nitrogen as adaptation response

**Figure 7:**
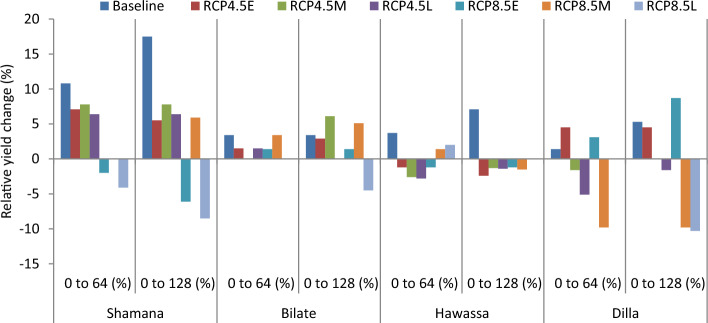
Change in grain yield (%) of shone variety in response to nitrogen application relative to control during early, mid and late-century across baseline, RCP 4.5 and 8.5 emission scenario at Shamana, Bilate, Hawassa and Dilla clusters, southern central Ethiopia.

**Figure 8:**
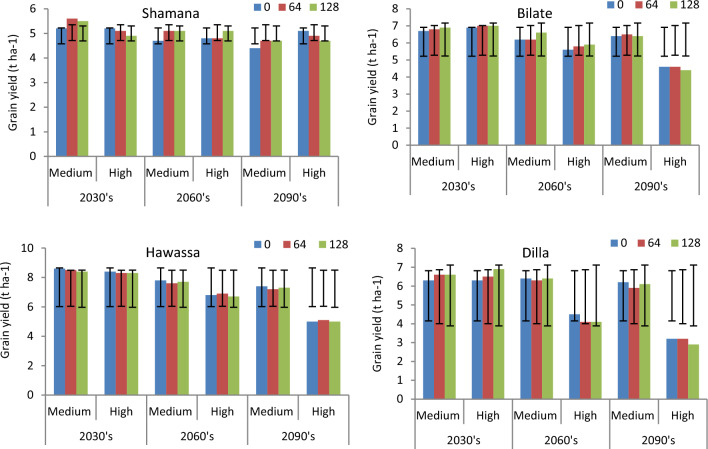
Maize yields (t ha^−1^) and error bars with standard deviation due to change in nitrogen rate under medium and high GHGs during early, mid and late century period across Shamana, Bilate, Hawassa and Dilla clusters.

The projection of maize yield (8.5  t ha^−1^) is highest at Hawassa due to 64 kg ha^−1^ nitrogen across medium and high GHGs emission scenario during early century. The lowest grain yield of 2.9 t ha^−1^ was projected in Dilla due to 128 kg ha^−1^ nitrogen across high GHGs emission scenario during late century. Considering yield changes, increasing nitrogen rate from 0 to 64 kg ha^−1^ resulted in 7.1, 7.8 and 6.1% increase of maize grain yield under medium GHGs emission scenario during early, medium and late century period, respectively compared with the control (0 kg ha^−1^ N) at Shamana. Increasing nitrogen rate further to 128 kg ha^−1^ resulted with 5.5, 7.8 and 6.4% increases in maize yield for the same period and GHGs emission scenario (Figs. 7 and 8). Thus, increasing nitrogen rate increased maize grain yield under baseline and medium GHGs emission scenario conditions during early, mid-term and late century period. However, under high GHGs emission scenarios, increasing nitrogen rate resulted in yield loss compared with control in early and late century period in Shamana.

At Hawassa, maize yield increased by 3.4% each when application rates are increased from 0 to 64 and 128 kg ha^−1^ during the baseline period. However, maize yields showed a decreasing trend during early, medium and late century period under medium and high GHGs emission scenarios. This depicts the vulnerability of the Hawassa cluster for small changes in emission of greenhouse gases. At Dilla cluster, increasing nitrogen rate from 0 to 64 and 0 to 128 increases maize yield by 1.4 and 5.3%, respectively during the baseline period. During early century period, increasing nitrogen application from 0 to 64 and 0 to 128 kg ha^−1^ increases maize yield by 4.5% each during medium GHGs emission scenario. At Dilla, increasing nitrogen application from 0 to 64 and 0 to 128 increases maize yield by 3.1% and 8.7%, respectively under medium GHGs emission scenario during early century period. The simulation result showed that increasing nitrogen application from 0 to 64 and 0 to 128 kg ha^−1^ results in yield disadvantage under high and medium GHGs emission scenarios during both mid and late century periods at Dilla cluster.

#### Mulching rate as adaptation response

**Figure 9:**
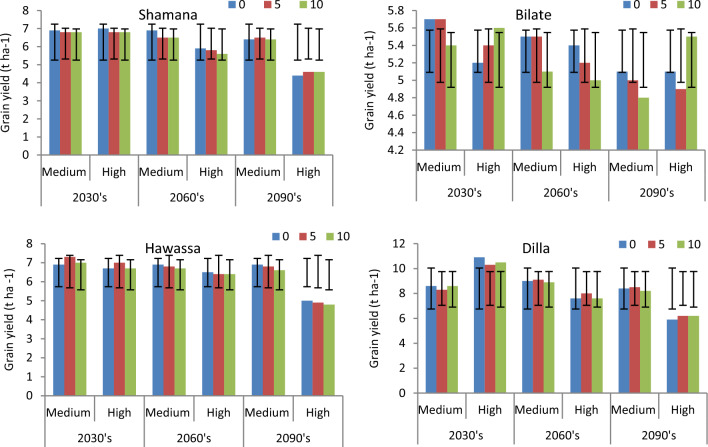
Maize yields (t ha^−1^) and error bars with standard deviation due to change in mulching rate under medium and high GHGs during early, mid and late century period across Shamana, Bilate, Hawassa and Dilla clusters.

**Figure 10:**
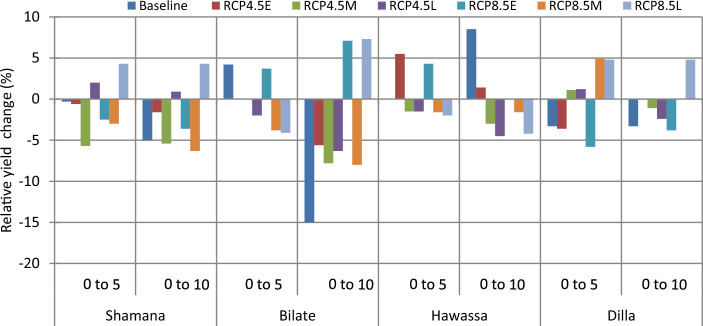
Change in grain yield (%) of shone variety in response to mulching relative to control during early, mid and late-century across baseline, RCP 4.5 and 8.5 emission scenario at Shamana, Bilate, Hawassa and Dilla clusters, southern central Ethiopia.

The projection of maize yield (12.1 t ha^−1^) is highest at Dilla due to mulching maize field with 5 t ha^−1^ across baseline scenario (Fig. 9). Considering yield changes, increasing mulching rate from 0 to 10 t ha^−1^ reduced maize yield under baseline, medium and high GHGs emission scenarios during early and mid-century at Shamana (Fig. 10). Moreover, maize yields increased by 2 and 4.3% under medium and high GHGs emission scenario, respectively due to application of 5 t ha^−1^ during late century period at Shamana (Figs. 9 and 10). Similarly, maize yields increased by 0.9 and 4.3% under medium and high GHGs emission scenario due to application of 10 t ha^−1^ during late century period at Shamana. At Bilate, increasing mulch rate to 5 t ha^−1^ increased maize yield by 4.2 and 3.7% during baseline and late century period under baseline and high GHGs emission scenarios, respectively. However, increasing mulch rate to 5 t ha^−1^ would result in negative yield change under high GHGs emission scenario during mid and late century period at Bilate. Further increase of mulching rate to 10 t ha^−1^ would inflict reduction in maize gain yield by 5.6, 7.8 and 6.3% in Bilate cluster during early, mid and late century periods, respectively under medium GHGs emission scenario. When mulching rate was increased from 0 to 10 t ha^−1^, maize yield would increase by 7.1 and 7.3% compared with baseline during early and late century period, respectively under high emission scenario in Bilate, but maize yield would decrease by 8% during mid-century period under medium GHGs emission scenario.

At Hawassa, increasing mulching rate from 0 to 5 t ha^−1^ increased maize yield by 5.5 and 4.3% under medium and high GHGs emission scenarios, respectively during early century period. Further increase of mulch to 10 t ha^−1^ resulted in 1.4 and 0% yield increase under medium and high GHGs emission scenarios in the same period. At Dilla, simulation of mulch application resulted in yield loss during baseline and early century period. For instance, application of 5 t ha^−1^ mulch resulted in decrease of grain yield of maize by 3.6 and 5.8% under medium and high GHGs emission scenarios, respectively during early century. However, application of 5 t ha^−1^ mulch resulted in 5 and 4.8% yield increases under high GHGs emission scenario during mid and late century, respectively. Similarly, application of 5 t ha^−1^ mulch resulted in 1.1 and 1.2% yield increases under medium GHGs emission scenario during mid and late century.

#### Simulation of integrated adaptation response

**Figure 11:**
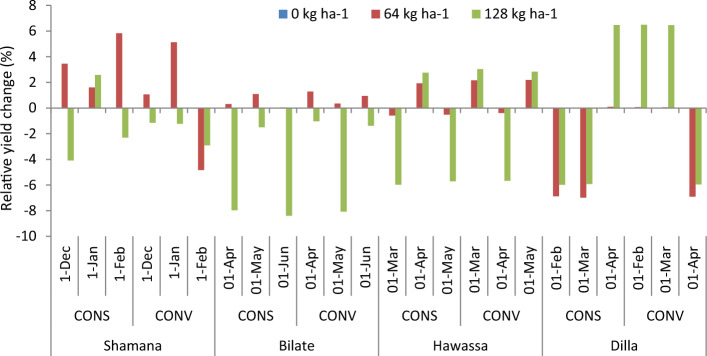
Percentage yield change from baseline in response to nitrogen, tillage and planting dates during mid-century across medium GHGs emission scenario for Shamana, Bilate, Hawassa and Dilla clusters.

**Figure 12:**
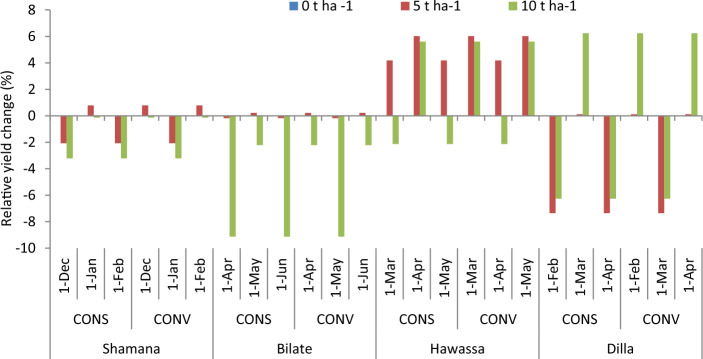
Percentage yield change from baseline in response to mulching, tillage and planting dates during mid-century across medium GHGs emission scenario for Shamana, Bilate, Hawassa and Dilla clusters.

**Figure 13:**
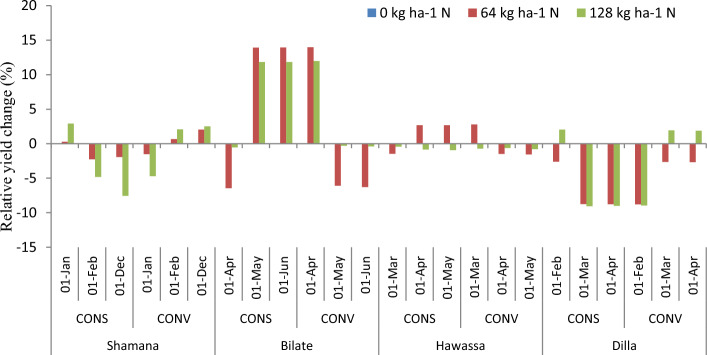
Percentage yield change from baseline in response to nitrogen, tillage and planting dates during mid-century across medium GHGs emission scenario for Shamana, Bilate, Hawassa and Dilla clusters.

Results of combination of adaptation options including planting early in December with conservation tillage and use of 64 kg ha^−1^ N provided 3.5% yield advantage over the control under medium GHGs emission scenario during mid-century period at Shamana (Fig. 11). Similarly, delaying planting to February, use of 64 kg ha^−1^ N and employing conservation tillage provides yield advantage of 5.8% over the control under medium GHGs emission scenario during mid-century period at Shamana. Conversely, delaying planting to February, use of high nitrogen rate (128 kg ha^−1^ N) and use of conventional tillage results in yield decline of 4.8% compared with the control. In Bilate, use of 128 kg ha^−1^ N resulted in yield change of − 1.1 to 8.4% compared with the control regardless of tillage and planting date adjustments. A combination of conservation tillage, April planting and 128 kg ha^−1^ N produced yield advantage of 1.9% compared with the control at Hawassa. If tillage is altered to conventional, then May planting suits as it renders 2.2% yield advantage in Hawassa under medium GHGs emission scenario during mid-century (Fig. 11). At Dilla, use of conservation tillage and 128 kg ha^−1^ N results in 6.5% yield advantage if planting date would be shifted from March to April. The advantage of applied adaptation options would be low in Bilate and Hawassa clusters during mid-century period which could be mainly due to recurrent moisture stress projected to occur in the clusters. Mulching 5 t ha^−1^ was projected to produce 4–5% yield advantage in Hawassa cluster during mid-century period regardless of changes in tillage or planting window (Fig. 12). Under high GHGs emission scenario during mid-century period, over 13.4% yield advantage was projected in Bilate cluster due to conservation tillage and late planting, which is June (Fig. 13).

**Figure 14:**
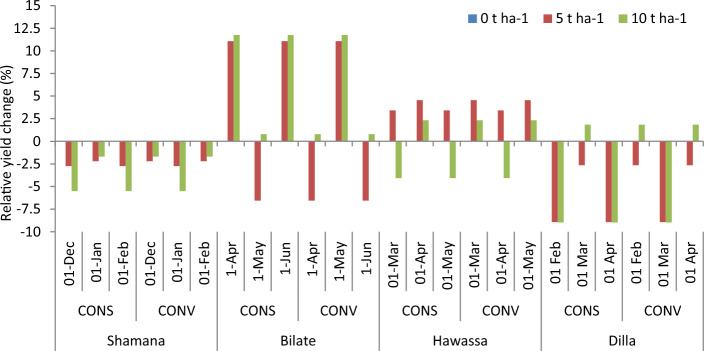
Percentage yield change of maize from baseline in response to mulching, tilage and planting dates during mid-century across medium GHGs emission scenario for Shamana, Bilate, Hawassa and Dilla clusters.

Intigrated use of conservation tillage, 5–10 t ha^−1^ mulch and April planting produced over 11% yield advantage in Bilate across high GHGs emission scenarios during mid-century period. Similar yield advantage could be obtained if tillage remained conventional and planting maintained in May so long as 5–10 t ha^−1^ mulch retained in the soil at Bilate (Fig. 14). In Dilla cluster, use of 10 t ha^−1^ mulch and conservation tillage and early planting (February) would result about 1.8% yield advantage compared with the control either in medium or high GHGs emission scenarios.

## Discussion

Considering a time period most relevant to large agricultural investments, which typically take 15–30 years to realize full returns^68^, impacts of adaptation strategies were closely looked at mid-century period in this study. During mid-century period, delaying planting to February, use of 64 kg ha^−1^ N and employing conservation tillage would provide yield advantage of 5.8% over the control under medium GHGs emission scenario at Shamana. At Bilate, conservation tillage and June planting produced over 13.4% yield advantage under high GHGs emission scenario during the same period. At Hawassa, mulching 5 t ha^−1^ is projected to produce 4–5% yield advantage during mid-century period regardless of changes in tillage or planting window. At Dilla, use of conservation tillage and 128 kg ha^−1^ N would result in 6.5% yield advantage if planting date would be shifted from March to April. The lowly performance of conventional tillage was attributed to moisture loss and erosion hazards particularly in Bilate and Hawassa clusters^69^ whereas better performance from conservation agriculture was due to conserving soil structure, storing soil carbon and nutrient which would ultimate;y enhance water retention^70^*.* Overall, future climate is expected to have a negative impact on maize yield as yields still go down with adaptations but the decrease is about less compared with conditions without adaptation strategies. This manifests the urgent need to adopt moisture conserving managment practices in all study areas.

Use of mulch and nitrogen has been proven responsive in highland Shamana cluster, mid-land Hawassa cluster and also in high rainfall receiving Dilla cluster across RCP 4.5 and RCP 8.5 during baseline and early century. The nutritional role of nitrogen on yield enhancement under future climate couldn’t be over emphasized, but the beneficial role of mulches could be due to enhancement of soil physico-chemical properties, suppression of weeds and reduction of evapo-transpiration that would ultimately lead to better maize productivity in dry land agriculture^71^. Conversely, lack of response to mulch application could be observed occasionally due to extensively low soil moisture and low nitrogen fertility as in Bilate cluster, and the humid climate of Dilla cluster where slow decomposition of already available mulch layers occur^72^. The variability in yield improvements due to nitrogen applications observed in the current study across clusters agreed with findings of Liu and Basso^73^ that attributed spatial variability in response of nitrogen strategies to differences in weather, soil biophysical and chemical properties of soils, and their interactions with management.

The climate in baseline and early century is projected to reward a better grain yield due to soil mulching. Similarly, increasing nitrogen rate increased maize yield under medium and high GHGs emission scenario across early and mid-century period. During late century period, positive increases from applied nitrogen were simulated only for moderate GHGs emission scenario and not for high emission scenario. The yield loss anticipated across most clusters under high GHGs emissions were attributed to likely enhanced rate of dvelopment and pollination due to high temperature stress under future climate^74^. According to this authors, high temperatures lessens the pollen viability, fertilization, and grain creation in maize^74^. This has been excerbated by frequent low moisture regimes which subsequently reduces nutrient uptake and response to nitrogen applications. Thus, despite benefiting from elevated (CO_2_) with increased use of N fertilizers, maize plants will be increasingly negatively impacted as temperature increases and/or if rainfall decreases under high GHGs emission scenarios. Previous research also showed both retention of harvest residue and N fertilization under RCP 8.5 would enhance maize yield^39^; however, also reported that increased CO_2_ concentration would enhance the labile C input to the soil, which may increase N_2_O and NH_3_ emissions through encouraged microbial activity^75^, which would probably hamper productivity. Hence the yield penalty remain high because the benefits of elevated CO_2_ were unlikely to offset the negative impacts of increasing temperature and variable rainfall.This calls for controlling GHGs emissions to enhance maize production and obtain maximum rewards from investment in adaptation strategies.

The current study calls for changing conventional tillage to conservation tillage in Shamana and Dilla clusters during baseline scenarios, and also starting early century at Bilate and Hawassa clusters. Changing tillage from conventional to conservation was found beneficial across most clusters under present and future climate scenario which agreed with Hemmat and Eskandari^76^ and Kephe et al.^16^ who attributed improvement in crop establishment, growth and yield due to enhanced soil water content under conservation tillage. Dong et al.^77^ associated higher yields under conservation tillage due to improvement of soil organic carbon and crop water use efficiency. Although the DSSAT model did not simulate any gains or losses in grain yield due to delayed planting at Hawassa and Dilla, there were positive yield gains in Bilate and Shamana. This is mainly because delayed planting avoids the high-risk periods of heat and drought stress in the growth period of the crop. This agreed with some authors who attributed the advantage of delayed planting to increased precipitation during late July to early August, which resulted in higher pollination rates and kernel numbers^65,66^. However, delayed planting previously recommended by Disasa and Yan^67^ for Hawassa cluster was not validated with multiple climate ensembles considered in this study.

The positive yield gains observed with 128 kg ha^−1^ nitrogen in Shamana, Bilate and Dilla under baseline and medium GHGs emission scenarios were in agreement with findings of He who recommended 150 kg N ha^−1^ in Canada based on the DSSAT model^13^ and Kassie et al.^59^ that indicated increasing nitrogen fertilizer rate of 60 kg ha^−1^ to increase maize yield by 78–89% in Ethiopia. The yield loss observed at Hawassa due to increased nitrogen use under future climate scenarios could be due to high nitrogen application despite low nutrient use efficiency leading to resources wastage, greenhouse gas emissions and nitrate leaching^13^ and has been attributed to the lower soil water regime after fertilization^75^. The current study also proves findings of Smith et al.^15^ who disclosed yield loss across high emission scenarios during late century despite use of alternative adaptation options.

The current study showed that most promising and least risky practices among simulated strategies would be enhancing integrated use of nitrogen and mulching across the clusters in combination with either tillage or planting date options. However, planting date and tillage interventions remained least promising and highly risky practices if not integrated with residue or nitrogen in order to adopt climate change in the studied clusters. Hence, nitrogen fertilization and residue mulching require suitable moisture regimes prior application. This necessitates integration of nitrogen or mulches with planting date or tillage in maize fields, which is vital to reduce higher emission of green house gases. Moreover, this study is limited to biophysical impact of only four adaptation options, and didn’t include bio-economic analysis. Similarly adaptation responses related to rotation, water harvesting, irrigation, phosphorus application or modification of cropping systems were not addressed. To generate other effective adaptation options that reflect local conditions, there is a need to understand the characteristics of the maize crop under simultaneous and relay intercropping with N-fixation legume cover crops and split applications of mineral fertilizer, which may be important in high rainfall cluster of Dilla as nitrate leaching could lead to low response of applied nitrogen. In low rainfall and warm clusters of Bilate and Hawassa, drought tolerant maize varieties, water-harvesting technologies (stone lines, tied ridges, *zaï* pits and contour ridges), and in low laying Bilate cluster, cultivars adapting heat stress may help reduce production losses. For all the clusters, accounting for P stress may help to reduce model uncertainty as maize crop responds to applied P in all clusters except Hawassa. There is a need also to explore supplemental irrigation as one of adaptation strategies where water can be availed through alternative sources particularly during critical periods of moisture stress to the crop. If the growth time of maize from silking to maturity could be prolonged and the accumulated temperature could be raised, which requires sufficient moisture during his stage, the dry matter accumulation of maize would effectively increase, which would have an obvious effect on maize yield.

## Summary and conclusion

The present investigation is undertaken to explore effects of adaptation responses including tillage, mulching, planting dates and nitrogen on productivity of maize variety using DSSAT model v 4.8. Impacts and adaptation options were evaluated using projections by 20 coupled ensemble climate models under two representative concentration pathways (RCPs) 4.5 and 8.5. It was found that farmers' yield is 46–54% of on-farm experimental yield, and in turn on farm experimental yield was 23.5–45.2% of the water limited potential yield in southern central rift valley of Ethiopia leaving 1.9 to 3.1 t ha^−1^ gap between research and farmers’ yield and 3.4–5.6 t ha^−1^ gap between water limited potential yield and farmers’ yield. Under RCP 4.5, maize grain yields changed by 1.4–5.7%, 1.2–3.1%, 0–1.2% and 1.4–1.6 due to conservation tillage at Shamana, Bilate, Hawassa and Dilla clusters, respectively. However under RCP 8.5, maize grain yields changed by 0%, 1.4 to 1.5%, −2 to 1.5% and − 2.3 to 1.6 due to conservation tillage in the same locations. Thus, there is reduced response by maize yield for tillage change at high emission scenario across most clusters. Changing planting dates forward (from January to February) changes maize yield by − 2 to 2% and 0 to 1.4% at Shamana during RCP 4.5 and baseline scenario, respectively. Delaying planting dates by a month (changing from January to December) increased maize yield by 0 to 1.8% across RCP4.5 at Shamana. Thus under high emission scenario, delayed planting would act as adaptation option but early planting did not at least in the study clusters. Planting date changes did not affect maize yield significantly (P < 0.05) at Hawassa and Dilla clusters under future climate.

Maize grain yields changed by − 8.5 to 17.5%, − 4.5 to 5.1%, − 2.3 to 7.1% and − 9.8 to 7.1% due to increased use of nitrogen compared with control (0 t ha^−1^) at Shamana, Bilate, Hawassa and Dilla clusters, respectively. Thus, better response is projected across highland and mid-altitude clusters of Shamana and Hawassa compared to low land clusters of Bilate and Dilla thereby reducing vulnerability of maize production to risks related to climate change in former agro-ecologies. Yield loss from application of nitrogen were evident across RCP 8.5 scenario which was mainly due to the lower soil water regime after fertilizer application. Thus, under high GHGs emission scenarios, increasing nitrogen rate increased risk of yield decline compared with control in early and late century, which necessitates controlling emissions to enhance maize production and maximize benefit from applied nitrogen fertilizers. At Hawassa, increasing mulching rate from 0 to 5 t ha^−1^ increased maize yield by 5.5 and 4.3% under medium and high emission GHGs scenarios, respectively during early century period. Moreover, application of 5 t ha^−1^ mulch resulted in 5 and 4.8% yield increases under high GHGs emission scenario during mid and late century, respectively at Dilla. Generally, maize grain yields changed by − 6.3 to 4.3%, − 15 to 7.7%, − 1.6 to 8.5% and − 5.8 to 4.8 due to the use of mulches compared with control at Shamana, Bilate, Hawassa and Dilla clusters, respectively. Yield loss from use of mulches were evident across RCP 8.5 scenario. Higher response due to applied mulches is projected at Bilate and Hawassa compared with the reponse in Shamana and Dilla, which shows that mulches would become important component of climate change response strategy.

The combined result of adaptation responses including February planting, use of 64 kg ha^−1^ N and conservation tillage provided yield advantage of 5.8% over the control under medium GHGs emission scenario at Shamana during mid-century period*.* At Bilate, conservation tillage and June planting produced over 13.4% yield advantage under high GHGs emission scenario during the same period. Mulching 5 t ha^−1^ was projected to produce 4–5% yield advantage in Hawassa cluster during mid-century period regardless of changes in tillage or planting window. Under high GHGs emission scenarios, over 13.4% yield advantage was projected in Bilate cluster during mid-century period due to conservation tillage and June planting, which is late planting for the cluster. A combination of conservation tillage, April planting and 128 kg ha^−1^ N produced yield advantage of 1.9% compared with the control at Hawassa during mid-term period under medium GHGs emission scenarios. At Dilla, use of conservation tillage and 128 kg ha^−1^ N would result in 6.5% yield advantage if planting date is shifted from March to April. In all the clusters, conservation tillage allowed better yield advantage if planting is delayed. Mulching was found to benefit maize production more in Hawassa and Bilate clusters than Shamana and Dilla, the former being high potential but moisture stressed compared with the later. Hence, altering planting date alone, integrating planting date with residue, tillage with nitrogen or planting date with tillage remained least promising and highly risky practices if not integrated with mulch application or nitrogen use in order to adopt climate change in the study clusters. Thus, most promising and least risky practice among simulated strategies would be amalgamation of nitrogen and mulching with either tillage or planting date options, and hence optimized agronomic crop management practices can be considered as effective adaptation strategies to climate change for maize production in southern central rift valley of Ethiopia.

## Data Availability

This study utilizes Coordinated Regional Climate Downscaling Experiment (CORDEX) reanalysis data Sponsored by the World Climate Research Program for daily baseline (1980–2010), and future climate of a 30-year window period representing climate projections for Representative Concentration Pathways (RCP) 4.5 and 8.5 during the near term (2011–2039), mid-term (2040–2069) and end-term (2070–2100) from contrasting multimodel ensembles of 20 Global Climate Models (GCMs) and Regional Climate Models (RCMs), which were downscaled to 44 km horizontal resolution for the Africa domain were accessed for each cluster from source http://www.csag.uct.ac.za/cordex-africa data base.

## Abbreviations

4.5E: Medium GHGs emission scenario during early century
4.5M: Medium GHGs emission scenario during mid-century
8.5E: High GHGs emission scenario during early century
8.5L: High GHGs mission scenario during late century
8.5M: High GHGs mission scenario during mid-century
4.5L: Medium GHGs emission scenario during late century
CO_2_: Carbon dioxide
CONS: Conservation tillage
CONV: Conventional tillage
GHGs: Green house gases
MSE: Mean square of error
N: Nitrogen
N_2_O: Nitrous oxide
NH_3_: Ammonia
P1: Thermal time from seedling emergence to the end of the juvenile phase
P5: Thermal time from silking to physiological maturity
PD: Planting date
RCP: Representative concentration pathway
RS: Residue retention
TL: Tillage

## Code availability

Code used for this study is available upon reasonable request from the corresponding author.
